# A Quantitative Description for Optical Mass Measurement of Single Biomolecules

**DOI:** 10.1021/acsphotonics.3c00422

**Published:** 2023-06-23

**Authors:** Jan Becker, Jack S. Peters, Ivor Crooks, Seham Helmi, Marie Synakewicz, Benjamin Schuler, Philipp Kukura

**Affiliations:** † The Kavli Institute for Nanoscience Discovery, University of Oxford, Dorothy Crowfoot Hodgkin Building, South Parks Rd, Oxford OX1 3QU, U.K.; ‡ Physical and Theoretical Chemistry Laboratory, Department of Chemistry, University of Oxford, South Parks Road, Oxford OX1 3QZ, U.K.; § Department of Biochemistry, University of Zurich, Winterthurerstrasse 190, Zurich 8057, Switzerland; ∥ Department of Physics, University of Zurich, Winterthurerstrasse 190, Zurich 8057, Switzerland

**Keywords:** mass photometry, polarizability, single molecule, label free, mass measurement

## Abstract

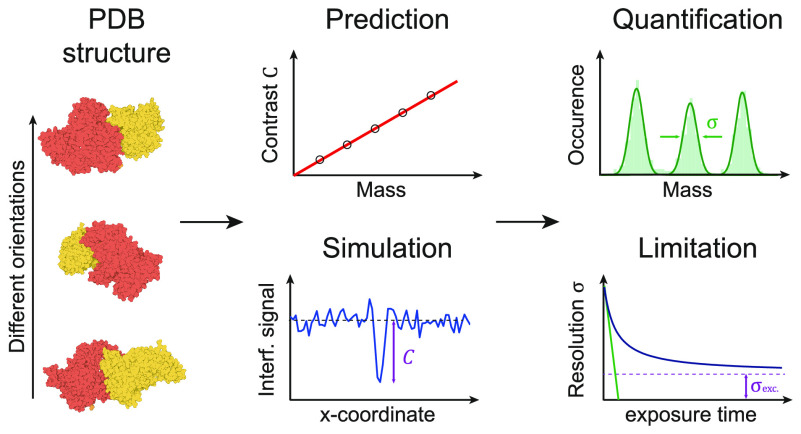

Label-free detection of single biomolecules in solution has been achieved using a variety of experimental approaches over the past decade. Yet, our understanding of the magnitude of the optical contrast and its relationship with the underlying atomic structure as well as the achievable measurement sensitivity and precision remain poorly defined. Here, we use a Fourier optics approach combined with an atomic structure-based molecular polarizability model to simulate mass photometry experiments from first principles. We find excellent agreement between several key experimentally determined parameters such as optical contrast-to-mass conversion, achievable mass accuracy, and molecular shape and orientation dependence. This allows us to determine detection sensitivity and measurement precision mostly independent of the optical detection approach chosen, resulting in a general framework for light-based single-molecule detection and quantification.

## 1 Introduction

**Figure 1:**
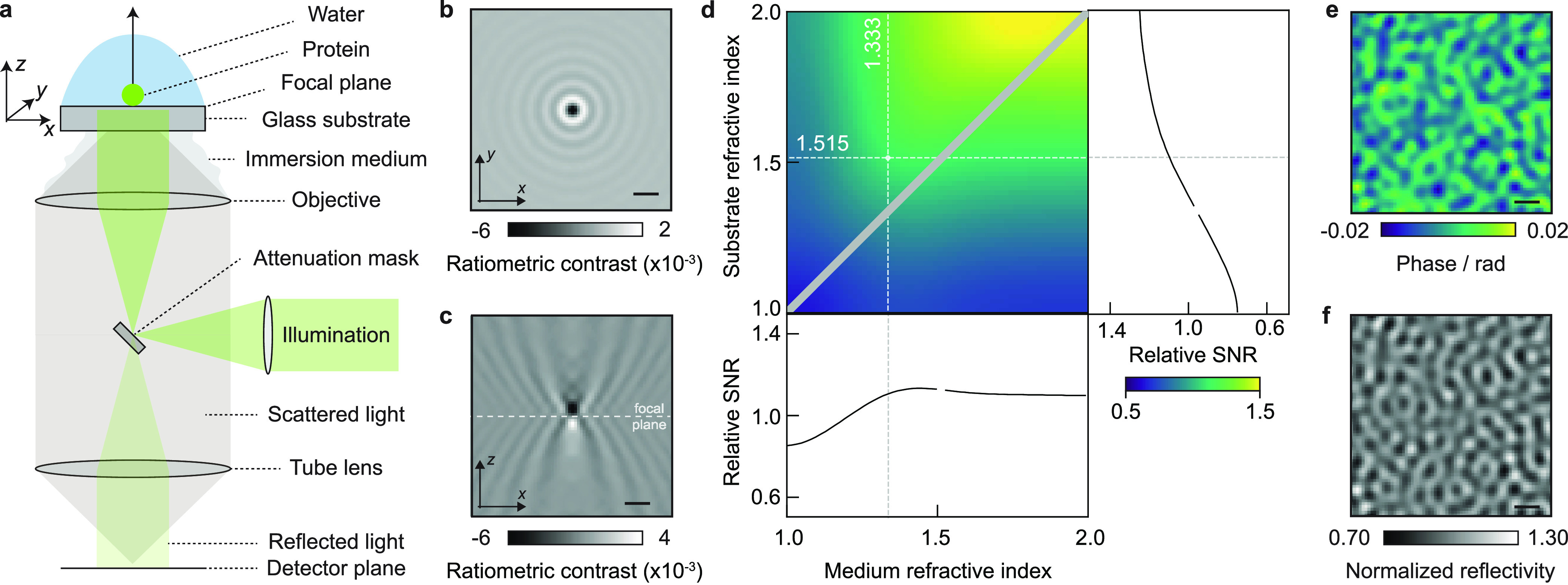
Fundamentals of image formation in a contrast-enhanced back reflection geometry. (a) Schematic of the simulated, widefield, MP setup, including an attenuation mask in the BFP of the objective lens, which selectively reduces light reflected from the glass coverslip. (b,c) Simulated ratiometric contrast for a single 24-mer of the small heat shock protein Hsp16.5 (*m* = 396 kDa) at an atomically flat glass–water interface. (d) Relative SNR when varying the refractive indices of the substrate and buffer medium. The diagonal indicates refractive index matching, where no reference field is available due to a lack of reflection. (e) Simulated phase retardation map at λ = 445 nm arising from nanoscopic roughness of glass coverslips on the order of ∼2 nm height variations over ∼100 nm (lateral) length scales. (f) Resulting speckle-like image using a 0.1% transmission mask, normalized to the expected reflectivity from a flat glass–water interface (see Section S6). Scalebars = 0.5 μm.

Recent developments in ultrasensitive light microscopy^1,2^ have enabled the quantification of biomolecular mass, charge, and size at the single-molecule level and in solution.^3−8^ In mass photometry (MP), light scattered from a protein when it binds to or moves along a glass coverslip in solution is detected together with partially reflected light from the glass–water interface (Figure 1a). MP has demonstrated both high mass accuracy and precision on the order of a few percent of the object mass, enabled by high-measurement precision at the single-molecule level.^3^ This has been achieved through selective attenuation of the reflected light using a mask in the back-focal-plane (BFP) of the optical system,^9^ coupled with averaging of detected photoelectrons by the imaging camera and post-processing of the raw images, enabling the detection and resolution of oligomeric states and protein complexes.^10^ As a result, MP can be used to quantify interaction affinities and kinetics,^11^ molecular organization,^12^ and for studies of biomolecular dynamics.^13^

The molecular mass, *m*, for unknown samples is inferred from the optical contrast of the molecule under investigation and an empirical scaling between the contrast and a species of known molecular mass. This relationship can be approximated by estimating the excess polarizability α using the refractive index of proteins *n*_p_ and assuming a spherical shape.^14^ In fact, a number of approaches have been reported recently to calculate the optical signal in interferometric scattering microscopy, which can be combined with such simplified models of biomolecules to predict images and expected optical contrast.^15,16^ Nevertheless, all models to date did not consider the atomic nature of biomolecules, making any attempts to compare experimental and theoretical results largely qualitative and unable to predict effects of molecular shape or orientation, for example. Given that the refractive index of a single protein is poorly defined, we thus lack a molecular-level description of light-based mass measurement and what properties define the limits and opportunities in measurement sensitivity, precision, and accuracy.

We thus set out to develop an approach capable of simulating images of individual proteins on a microscope cover glass in an MP instrument coupled with an explicit atomic description of molecular polarizability and thereby explore key aspects such as (1) to which degree optical contrasts reported to date experimentally match those predicted by theory. (2) How the measured signal depends on the molecular shape and orientation, thereby informing on the ultimately achievable mass accuracy and resolution. (3) The current and likely future limits on measurement sensitivity and resolution for light-based single-molecule characterization for MP and beyond.

## 2 Results

Our model simulates an experimental setup based on plane wave illumination, a (simplified) single lens high numerical aperture (NA) objective for light delivery and collection that is refractive index matched to the sample interface (here, glass; *n*_g_), and a protein embedded in a medium of refractive index *n*_m_ (e.g., water). A spatial mask is used to selectively attenuate light reflected from the coverslip substrate, which increases the optical contrast, simplifies the accurate determination of the focal position for maximum contrast, and allows for higher illumination power, given limited camera full well capacities (Figure 1a).^11^ We mathematically model the influence of the imaging system as the following convolution operation, defined as , with an amplitude point-spread-function (APSF)^17^*h⃗*
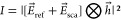
1yielding the detectable intensity *I*, with  and  being the reflected and scattered electric fields, respectively. These are directly linked to the illumination  through
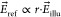
2
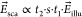
3where *r* and *t* are the Fresnel coefficients for reflection and transmission,^18^ and the subscripts indicate transmission of  from glass → water (*t*_1_) or that of the scattered field  from water → glass (*t*_2_). Note that the influence of the attenuation mask is realized by propagating both,  and , into the BFP of the objective lens, where they are being multiplied by a circular mask (corresponding to an effective NA, of 0.58) with a given transmission strength |τ|^2^ (1%; unless otherwise stated), motivated by experimental parameters.^9^ The scattering coefficient *s* scales with the polarizability α of the protein, which in the Rayleigh regime^14^ can be approximated to be proportional to the particle volume
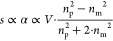
4

Note that our description of image formation mainly differs from experimental MP setups through non-scanned illumination, which is experimentally used to allow for widefield illumination without being limited by speckle-artifacts and to minimize the spatial extent of the molecular point-spread function.^3^ Further details on the employed theoretical model, such as including the near-field effects of protein scattering at a refractive index interface, high NA focusing effects, and phase aberrations due to imaging into a layer of different refractive index (buffer vs glass) are given in Sections 4.1 and S1–S8.

We begin our numerical investigation with Hsp16.5, a highly symmetric small heat shock protein, which forms spherical 24-mers of *m* = 396 kDa adsorbed on a glass substrate covered by water to enable direct comparison with early experiments.^9^ In a first iteration, we approximated the protein as a sphere of radius 5.6 nm and refractive index *n*_p_ = 1.480.^19^ The substrate refractive index was chosen to be *n*_g_ = 1.515 as typical for the borosilicate microscope cover glass used in MP^18^ but assumed to be atomically flat for simplicity. Experimentally, the limited full well capacity of CMOS imaging sensors requires both spatial and temporal summing of detected photoelectrons to reduce shot noise-induced background fluctuations and thereby optimize the attainable signal-to-noise ratio (SNR), taken as the ratio between the maximum signal amplitude introduced by the protein and the unknown background variations. In our simulations, we can take advantage of, in principle, unlimited full well capacities of the (virtual) camera pixels, which simplifies the image generation. The original experiments on Hsp16.5 used 2 × 2 spatial binning, corresponding to 60 nm/pixel at the reported magnification,^9^ followed by summing photoelectrons from 100 subsequent images, leading to a total of 10^7^ detected photoelectrons per spatio-temporally binned camera pixel.

The quantity typically used to report the optical contrast in MP is that of the ratiometric contrast *C*
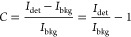
5which is the relative difference between the measured intensity with (*I*_det_) and without (*I*_bkg_) the scatterer.^9^ At the experimental illumination wavelength of λ = 445 nm, the simulated (here, without shot noise; details on simulation parameters are given in Section 4.2) ratiometric contrast of *C* ∼ 0.62% is close to the experimental result of 0.6%, as is the appearance of Airy rings arising from plane wave illumination (Figure 1b). Note that the maximum contrast does not coincide with the nominal focus position (Figure 1c), instead requiring a displacement of the sample by ∼190 nm along the optical axis to optimize the phase difference between scattered and reflected light by tuning the Gouy phase.^20^ Small particles further away from the plane being imaged are typically not detectable in MP due to their weak scattering and rapid diffusion compared to the camera exposure time, yielding an even smaller signal per pixel as it is distributed over a larger spatial area once it has reached the image plane. Our simulated results suggest that our model produces image contrasts in good agreement with experiments, where the contrast is optimized by maximizing the (spatial) standard deviation of the glass roughness. At the same time, this agreement is to be taken with care, given the rather arbitrary definition of particle radius and refractive index.

To explore the dependence of the image contrast on the refractive index of both the medium and the substrate, which in principle are tuneable away from that of water (*n*_m_ = 1.333) and borosilicate glass (*n*_g_ = 1.515), we varied both parameters and evaluated the achievable SNR for a constant power density incident on the sample. While increasing both refractive indices for a fixed refractive index of the protein (*n*_p_ = 1.46), which we assume to be non-tuneable, leads to a modest increase in the achievable SNR (Figure 1d). Nevertheless, these considerations may be of interest for measurements in environments of different refractive indexes to water, such as those containing glycerol or sucrose, even though such refractive index tuning is unlikely to have a dramatic impact on the ultimate performance of light-based single-molecule detection and mass measurement.

MP requires the removal of a static background image, which is the main reason for generating ratiometric images.^1,2,21^ This background resembles a speckle pattern generally attributed to nanoscale roughness of a microscope cover glass. Indeed, a recent report successfully correlated nanoscale roughness measured by atomic force microscopy with the corresponding image contrast.^22^ Using the reported surface roughness parameters in terms of lateral (∼100 nm) and vertical (∼2 nm) dimensions, we simulated the resulting phase retardation ψ of the reflected field, through
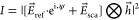
6

Note that ψ describes an effective phase change, which also accounts for the phase delay of the electric field that transmits through the glass–water interface and eventually leads to protein scattering. To obtain the speckle-like appearance in our simulation, we create a spatial array of randomly chosen numbers drawn from a uniform distribution , which is then spatially low-pass filtered to yield a near diffraction-limited speckle pattern. Overall, this yields a surface height map Δ*h* (varying between ±0.8 nm),^22^ which can be converted into the relevant phase distortion (Figure 1e) via
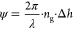
7

Including this phase variation in our model predicts an image contrast in agreement with experimental results (Figure 1f),^9^ effectively resulting in a locally varying reflection coefficient *r*.

**Figure 2:**
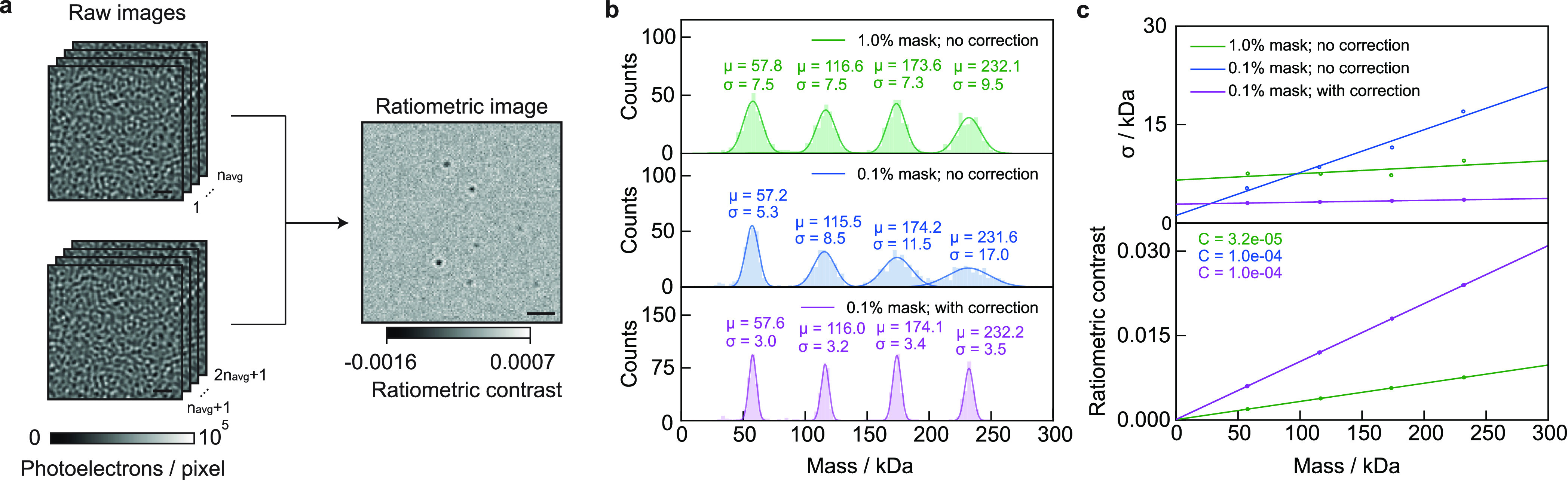
Simulation of protein landing events and resulting mass distributions as a function of mask strength. (a) Two consecutive sets of *n*_avg_ frames are averaged before computing the ratiometric image, revealing individual proteins landing at different positions as a function of time (scale bars: 1 μm). (b) Mass histograms for four oligomeric states of a protein simulated as spheres of radius 3.8 nm and refractive index 1.46 (representing BSA) using different mask strengths and local reflectivity correction.^9^ In all cases, the power incident on the detector was kept constant. (c) Standard deviation of the fitted distributions (top) and ratiometric contrast (bottom) as a function of protein mass.

Given that we can now produce raw images of both microscope cover glass and of single proteins with appropriate optical contrasts and spatial patterns, we can simulate a standard MP experiment (a general description of the typical experimental routine and materials is given in Sections 4.3 and 4.4). Here, a cleaned glass coverslip is usually covered by a dilute solution of biomolecules of interest, which bind non-specifically to the glass surface over time. These binding events are best visualized by averaging a series of camera frames of the glass surface and computing the relative difference between consecutive sets as a function of time^3^ (see Section 4.5). In this way, individual molecules are revealed, even though their contrast is much smaller than that generated by the glass roughness (Figure 2a).

Aside from detection sensitivity, the key performance parameter that determines the utility of MP is the achievable mass resolution, which originates in the measurement precision achievable on a molecule-by-molecule basis. Using illumination power (1% mask: 0.025 MW cm^–2^; 0.1% mask: 0.25 MW cm^–2^), wavelength (445 nm), and 10^7^ detected photoelectrons per pixel (Section 4.2), subsequent to temporal and spatial binning, we find peak widths similar to optimal experimental results on the order of σ = 8–9 kDa (Figure 2b, top). Using a mask with lower transmission in combination with higher illumination power leads to reduced peak widths at low mass but a broadening as mass increases. This is caused by the influence of the glass roughness on the ratiometric contrast, which now not only depends on the scattering coefficient *s* but also on the locally varying reflectivity *r* of the glass–water interface (neglecting the purely scattering term)
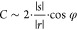
8with cos φ describing the phase difference between  and  at the detector plane. The glass roughness now results in the ratiometric contrast being dependent on where a particle lands on the glass coverslip. In the simulation, this broadening can be minimized by performing a correction step (based on ref (23)) by multiplication with , as this removes the dependence on *r*
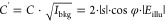
9

Note that applying eq 9 does retain most (∼99%) of the overall landing events, while maintaining their respective landing coordinates (∼91% matching in *x*, *y*, and frame number; see Section S17).

Applying this correction results in σ < 5 kDa peak widths but requires a homogeneous illumination field that is non-trivial to achieve in practice and the correction neglects an (unknown) phase contribution from the glass coverslip, which, in principle, results in residual broadening (see Section S6). In all cases, we observe behavior that agrees with expectations based on the selective reduction of reflected light by the transmission mask. A ten-fold reduction in reflected light is expected to increase the optical contrast by 10^1/2^, which would result in a 3.2-fold reduction in peak width (assuming a constant photon flux reaching the detector), comparable to our results (Figure 2c, top). Similarly, the concomitant increase in the contrast-to-mass conversion factor is also confirmed by our simulations (Figure 2c, bottom). These results validate the potentially high mass accuracy of MP, while assuming a perfectly spherical scatterer.

**Figure 3:**
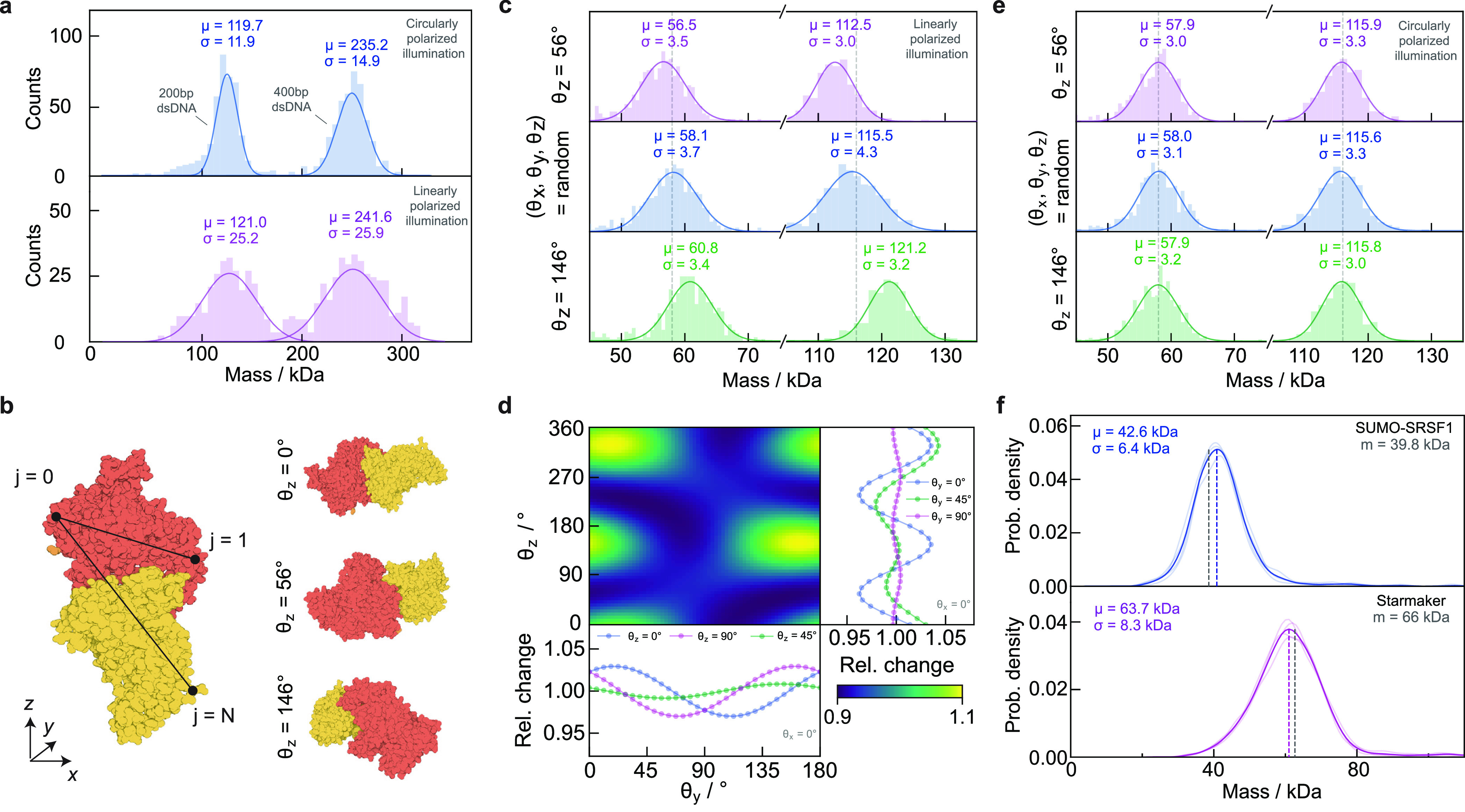
Dependence of mass measurement on biomolecular shape and illumination polarization. (a) Experimentally observed mass distributions for dsDNA illuminated with circularly (top) and linearly (bottom) polarized light. (b) Modeling the shape and orientation of a protein, here the dimer of BSA (PDB ID 3V03), by computing the corresponding polarizability tensor. (c) Mass histograms for a simulated landing assay where all BSA monomers (or dimers) land with the same (fixed) orientation (θ_*z*_ = 56°; θ_*z*_ = 149°), or with random orientations, while being illuminated with linearly polarized light. (d) Relative change of the ratiometric contrast for the BSA dimer for different orientations (changing θ_*y*_ and θ_*z*_) relative to the incident polarization. (e) Same simulation as in (c), except for circularly polarized illumination. (f) Kernel density estimation (Gaussian; bandwidth = 2 kDa) of experimentally measured distributions of partially (SUMO-SRSF1; 4 repeats) and fully disordered (Starmaker; 4 repeats) proteins. The vertical dashed lines represent the mass inferred from the measurement (colored) and the expected mass (gray).

To explore the effects of molecular shape and orientation beyond the spherical model, we chose double-stranded DNA (dsDNA), which effectively forms linear rods for a few hundred base pairs and below due to the persistence length of DNA.^24^ We performed MP measurements of a mixture of dsDNA with different numbers of base pairs (Section 4.6) while illuminating the sample with circularly polarized light (Figure 3a, top) and found no significant effect of the elongated shape of the DNA molecule on the ratiometric contrast when compared to proteins of similar mass in terms of peak widths (simulated results, assuming a perfectly rod-like shape, indicate the same and are shown in Section S14). The slight broadening for 400 bp DNA likely stems from the fact that the length of the DNA (∼136 nm) is no longer negligible compared to the diffraction limit, which can lead to the interferometric signal being spread over a slightly larger PSF and thus lower contrast, ultimately leading to peak broadening, an effect that becomes worse for longer DNA (see Sections 4.6 and S14). In principle, however, the enlarged PSF could be analyzed in a way to yield additional information on the size of the scatter, as shown in the supplement of Lee et al.,^25^ where this was done to infer the orientation of gold nanorods. When performing the experiment with linearly polarized light (Figure 3a, bottom), we found a 2-fold increase in peak width, which stems from the underlying variability of contrasts measured on a molecule-by-molecule basis (also see Section S14). These results suggest that scatterer shape and orientation can play an important role when employing linearly polarized illumination coupled with fixed molecular orientations. At the same time, it shows that the use of circularly polarized light makes MP essentially insensitive to molecular shape.

While folded proteins do not exhibit the degree of anisotropy as short DNA strands, they are also not spherical, especially in the context of oligomerization. We therefore turned to a recently reported approach^26^ using atomically resolved protein structures to compute the polarizability tensors of proteins from pairwise distances of all atoms in the molecule reported in the respective PDB structure (Figure 3b, left; also see Sections S7 and S8). The resulting polarizability tensor is a 3 × 3 matrix that encodes the anisotropic scattering of a particle as it connects any specific illumination direction with the scattering in all directions (denoted by *x*, *y*, and *z*; defined by the coordinate frame given in the PDB file)
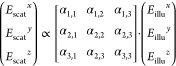
10

Note that any orientation of the protein can be achieved by multiplying the corresponding rotation matrices ***R*** = ***R***_***z***_·***R***_***y***_·***R***_***x***_ to determine the polarizability tensor **α**_rot._ in the new reference frame^27^
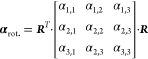
11In the first instance, we simulated a series of landing events, where we fixed the orientation of BSA (major axis misaligned: θ_*z*_ = 56° or aligned: θ_*z*_ = 56 + 90° = 146°; with respect to the linearly polarized illumination), meaning that all landing events have the same, fixed, orientation and compared the resulting mass distributions to those obtained from randomly oriented molecules (with θ_*x*_, θ_*y*_, and θ_*z*_ chosen such that the orientation sampling is uniformly distributed in 3D), for both monomers and dimers (Figure 3b, right). We find significant deviations from the nominal mass in both cases, on the order of 4% of the expected mass for the monomer and dimer (Figure 3c). A deviation of 4% amounts to the maximum observed difference when sampling the full range of possible protein orientations (Figure 3d, with varying θ_*y*_ and θ_*z*_). Repeating the simulation for BSA with circularly polarized light (Figure 3e) exhibited a drastically reduced dependence on protein orientation upon landing, now amounting to ≪1% of protein mass. While the deviations observed for linearly polarized illumination lead to broadening of individual mass peaks, mass photometers reported to date largely rely on circularly polarized light,^3^ making these measurements basically insensitive to protein shape. Note that the simulated protein mass for BSA of 58 kDa is lower than the mass based on its amino acid sequence (66 kDa) because the available PDB structure does not contain all atoms. When using the structure of BSA predicted by AlphaFold^28^ (UniProt P02769), we find excellent agreement between the mass inferred from our simulation (64.3 kDa; shown in Section S13) and the AlphaFold mass (64.4 kDa).

**Figure 4:**
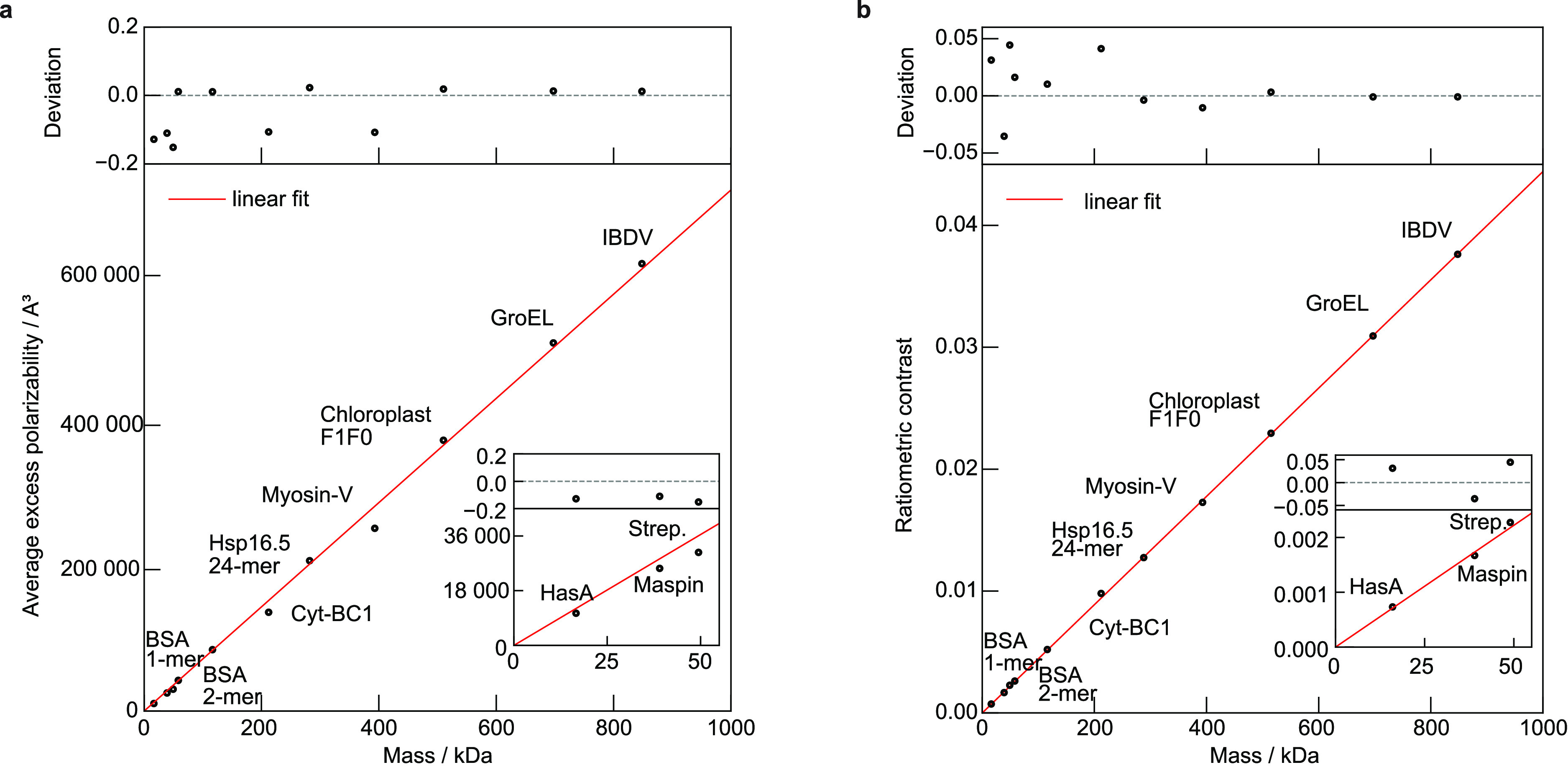
Mass scaling with molecular polarizability and image contrast. (a) Average excess polarizability and (b), calculated ratiometric contrast for proteins of mass 10–1000 kDa. Slope of linear fit (red line): 724 Å/kDa (a) and 4.4 × 10^–5^/kDa (b). PDB IDs: HasA = 1B2V; Maspin = 1XQJ; Strep. = 4BX6; BSA = 3V03; Cyt-BC1 = 1BE3; Hsp16.5 = 1SHS; Myosin-V = 2DFS; Choroplast F1F0 = 6FKI; GroEL = 1GR5; IBDV = 2GSY.

In addition to protein orientation, the degree to which a protein is folded could also have a substantial effect on the relationship between optical contrast and mass through various factors such as amino acid density or the association of water and counterions, all of which affect the molecular polarizability. We therefore turned to partially and fully unfolded proteins and compared the measured mass using folded proteins as a mass calibrant to the expected mass (Section 4.7). The small ubiquitin-related modifier (SUMO)-tagged serine- and arginine-rich splicing factor 1^29^ (SUMO-SRSF1) is a 39.8 kDa protein composed of an 11 kDA SUMO-tag, two 8–9 kDa structured domains (RRM1/2), and three intrinsically disordered domains totaling 10–11 kDa, making it 25% disordered by mass. The SUMO tag is a N-terminal carrier protein that promotes protein folding and stability, allowing for easier production of the desired protein.^30^ Using a folded, oligomeric protein as a calibrant (dynamin-1 ΔPRD), we obtain a mass of 42.6 ± 0.64 kDa (Figure 3f, top; magenta vertical dashed line), in good agreement with the predicted mass (gray vertical dashed line) and within the error found for various folded proteins (Figure 4b). We then turned to Starmaker, a 66 kDa, fully disordered protein.^31^ Due to its high negative overall charge, Starmaker does not bind adequately to standard microscope cover glass. We therefore functionalized the cover glass with (3-aminopropyl) triethoxysilane (APTES) to create a positively charged surface, obtaining a mass of 63.7 ± 0.4 kDa (Figure 3f, bottom; blue vertical dashed line), again in excellent agreement with the expected mass (gray vertical dashed line). In addition, in both cases we do not see any significant increase in the peak widths compared to folded proteins of similar mass.

We can now explore the previously reported linear relationship between optical contrast and mass for a variety of proteins, using random orientations for landing events and circularly polarized illumination as in the experiment. We find that the resulting (average) excess polarizability^16^ scales linearly with the respective molecular mass, derived from the amino acid sequence in the respective PDB entry (Figure 4a; the respective PDB IDs are given in Section 4.8, Table 1), with the polarizability change per mass δα
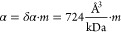
12which is slightly larger than that computed from bulk refractive index measurements^4^ (460 Å^3^ kDa^–1^). This deviation might be (partially) explained by including the factor *n*_m_^2^ into the absolute polarizability value, whose definition depends on how the scattering process is introduced in the corresponding calculation. When computing α for a range of proteins, some exhibit substantial deviation (>15%) from the expected linear relationship, especially below 200 kDa. To determine whether these variations in polarizability are reflected in real MP measurements, we used the full polarizability tensor model to calculate the respective ratiometric contrast from simulated landing assay movies as a function of protein mass. We found a linear relationship, this time with an root-mean-square (rms) error of 2.4% and improved agreement above 200 kDa (Figure 4b), both of which agree well with experimental results.^3,9^ The most likely reason for this improvement is that the average of the polarizability tensor does not report the scattering response to circularly polarized light but rather is an average measure of the particle scattering strength.^26^ Note that by theoretically establishing the linear relationship between protein mass and optical contrast, we are now able to move away from the simplified description using relatively poorly defined parameters, such as the refractive index of a single-molecule *n*_p_ and its radius (implicitly assuming a spherical shape), toward a more proper characterization based on the structure of the protein. We emphasize that we used the expected mass as calculated from the atomic positions in the respective PDB file rather than the nominal protein mass for all calculations, meaning that most proteins are found below their mass expected from their amino acid sequence.

Equipped with a realistic and quantitative description of the biomolecular polarizability and image formation, we can now deduce a general equation of the achievable SNR as a function of the key experimental parameters. First, we derive the number of detected, scattered, photons (*N*_sca_) per pixel and exposure time as (for the full derivation, see Section S10)
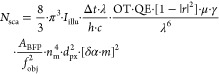
13with the following parameters defined in SI base units: *I*_illu_: illumination intensity [W/m^2^]; Δ*t*: effective exposure time per image [s]; λ: laser wavelength [m]; *h*: Planck’s constant [J s]; *c*: speed of light [m/s] OT: optical throughput; QE: quantum efficiency; |*r*|^2^: reflectivity of the glass–buffer interface; μ = sin^–1^(min[NA/*n*_*i*_,1])/π: collection efficiency of the detection objective; *d*_px_: effective pixel size in sample space [m]; *A*_BFP_ = π·*f*_obj_^2^·(*n*_*i*_·*n*_m_/*n*_g_)^2^: area of the accessible BFP, limited by the critical angle [m^2^]; γ: enhancement factor due to aplanatic factor and scattering beyond the critical angle (e.g., γ ∼ 1.58, for a 1.42 NA oil-immersion objective; see Sections 4.9, Table 2 and S10); *f*_obj_: focal length of detection objective [m]; *n*_m_: refractive index of buffer medium; *m*: mass of protein [kDa]; δα: polarizability change per kDa, i.e., the slope in Figure 4a.

The detected, reflected, number of photons *N*_ref_ (per pixel and exposure) is similarly given as
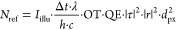
14with |τ|^2^ indicating the (power) transmission coefficient of the mask in the BFP. The shot-noise limited SNR in terms of the ratiometric contrast follows then as (see Section S10)
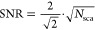
15with the factor of 2 originating from the interferometric nature of the signal and the  from the comparison of two subsequent sets of frames, required to remove the static background and form the ratiometric image. This can be converted into an equivalent expression for the detection limit of MP, i.e., the smallest detectable mass *m*_q_ at a certain SNR level. For this, we set SNR = *q* and solve for *m*
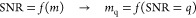
16with *f* denoting a functional dependency. Similarly, we can define a measure describing the lowest achievable mass resolution σ_m_, the Quantum-Cramer–Rao-Lower-Bound (QCRLB) as defined in ref (32)
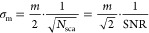
17

Note that the QCRLB relates to the uncertainty introduced by the quantum nature of light itself, i.e., represents a fundamental limit that almost all optical measurement techniques will obey.

We also find that this optimum achievable mass resolution is directly linked to the SNR-equivalent mass, with  (see Section S10 for the derivation)
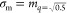
18meaning that the highest attainable mass resolution is equivalent to the mass that achieves an  in the shot-noise limited regime.

Given that most of the parameters are essentially fixed in realistic experimental scenarios, or only vary marginally as a result of the details of experimental implementation, we can simplify these expressions to depend only on some key experimental details, specifically illumination power, exposure time, wavelength, and molecular mass (in kDa).
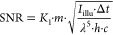
19with
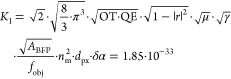
in SI units of m^4^/kDa, assuming OT = 0.8; QE = 0.70; |*r*|^2^ = 0.004; |τ|² = 0.02; *f*_obj_ = 3 mm; δα = 724 Å^3^/kDa; *n*_m_ = 1.333; μ = 0.40; γ = 1.58; *A*_BFP_ = 50 mm^2^; *d*_px_ = 80 nm. Yielding an SNR ∼ 21; *m*_*q*=3_ ∼ 9.5 kDa and σ_m_ ∼ 2.2 kDa for *I*_illu_ = 0.1 MW cm^–2^, Δ*t* = 100 ms, λ = 445 nm, and *m* = 66 kDa.

**Figure 5:**
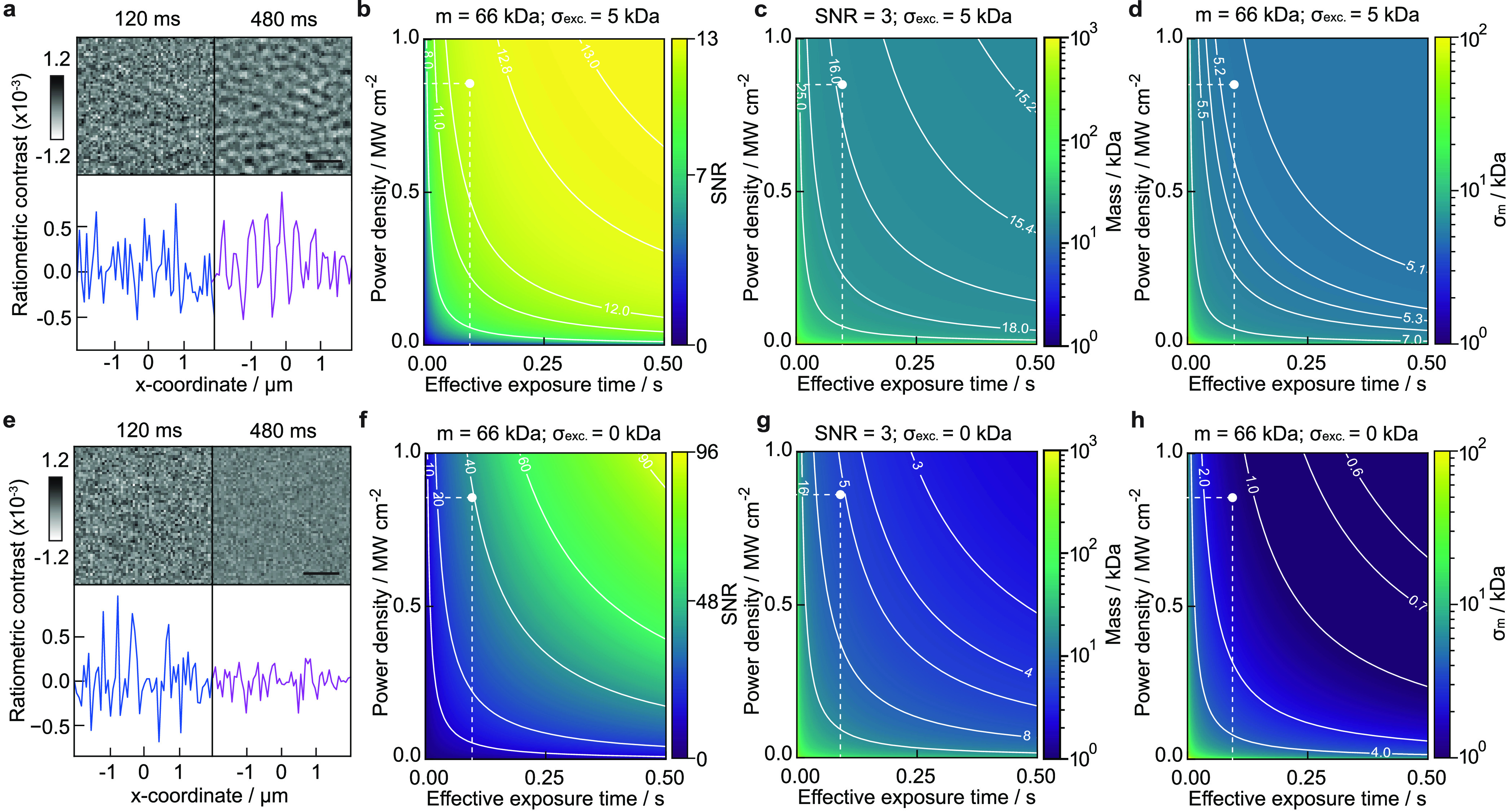
Current and future limits of optical mass measurement of single biomolecules. (a) Experimental ratiometric images of buffer medium only (scalebar = 1 μm) for different integration times. (b) Achievable SNR when detecting the BSA monomer (*m* = 66 kDa). (c) Smallest detectable mass *m*_*q*=3_ at SNR = 3. (d) Mass resolution σ_m_. All given as a function of effective exposure time and illuminating power density in the presence of excess noise on the order of 5 kDa. (e–h) Same (simulated) images and dependencies for purely shot noise limited performance. The white dots indicate experimental parameters from ref (3).

Experimental images, however, including those consisting of buffer medium only, or even ultrapure water, reveal a dynamic, speckle-like background at high imaging sensitivity that cannot be removed by temporal averaging or attributed to sample drift, here shown by comparing 120 and 480 ms averaging time, plateauing at a contrast on the order of a 5 kDa protein (Figure 5a). This background is likely the current limiting factor to both improving mass resolution and the absolute detection limit of MP and is close to that reported recently using machine learning.^33^ We currently have no clear explanation as to the origin of this additional noise source. As a first attempt we include it into our (SNR-) model, by adding it in quadrature to the shot-noise variance and find for the SNR including additional excess noise SNR_exc._ (with σ_exc._ being the added variance in terms of photon counts)
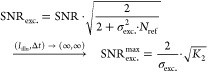
20and the constant
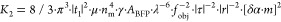
Note that SNR_exc._ naturally results in shot-noise limited performance for σ_exc._^2^ = 0 and yields a finite, maximum SNR_exc._^max^. The added influence of σ_exc._ has an overall impact on key performance parameters for protein detection and characterization, such as the achievable SNR (Figure 5b; with SNR_exc._^max^ ∼ 13.2), the lowest mass detectable (Figure 5c), and the achievable mass resolution (Figure 5d).

We can now compute theoretically achievable performance in terms of SNR, mass resolution, and detection limit (assumed for SNR = 3) both in the presence (Figure 5a–d) and absence (Figure 5e–h) of a non-shot noise contribution amounting to 5 kDa rms as currently observed experimentally. For realistic simulations, we find good agreement with previous reports,^3^ such as an SNR of ∼12.5 for BSA at an exposure time of 100 ms and 0.85 MW cm^–2^. Similarly, the recently reported SNR of 1.4 for a 9 kDa protein^33^ appears realistic, given that we find *m*_*q*=1_ ∼ 5.2 kDa with and ∼1.7 kDa without the additional baseline noise (for the same exposure time and illumination power density; see Section S16). Overall, our simulations in the absence of excess noise demonstrate that significant improvements in performance are still achievable with realistic illumination power (∼1 MW cm^–2^) and exposure times (<s), such as 1 kDa mass resolution and few kDa detection sensitivity, even in the absence of advanced image processing.

## 3 Discussion

We have presented a numerical approach that enables us to compute the optical contrast generated by individual biomolecules (smaller than the simulated pixel size) based on their atomic structure, orientation, and shape. Our results compare well with experimental data obtained by MP, suggesting that our model is indeed quantitative and asserting that there are no major (unknown) physical effects contributing to the current performance, which are not part of our theoretical description (such as Brownian motion or incomplete immobilization). We find a clear dependence of optical contrast on molecular shape in extreme cases such as short DNA strands illuminated by linearly polarized light, that weakens for folded proteins, and is essentially eliminated when using circular polarization. The predicted mass accuracy on the order of 2.4% rms matches that observed experimentally (2%), as does the computed dependence of optical contrast on the strength of the attenuation mask used. Our results on intrinsically disordered proteins support the hypothesis that the major determinants for the molecular polarizability are the constituent amino acids. In terms of achievable sensitivity, we present evidence for a dynamic, speckle-like background with a signal magnitude comparable to a 5 kDa protein. This background currently limits the ultimately achievable detection sensitivity and also affects the achievable mass resolution. The resolution is further affected by the static speckle-like background caused by microscope cover glass roughness, although it can be (partially) corrected for computationally.

Our results provide a quantitative framework for rationalizing label-free optical detection of single biomolecules. Polarizabilities, realistic incident power densities, and detection efficiencies effectively define achievable detection sensitivity and measurement precision at the single-molecule level, which translates into mass resolution. We emphasize that these relationships are independent of the optical approach, whether using total internal reflection^5^ or scattering from nanochannels.^4^ While there will be subtle differences in the achievable power densities and detection efficiencies, the presented limits are likely to be representative of what can be achieved in terms of mass measurement using light-based detection of single biomolecules. In all cases, when comparing experimental with these theoretical results, it is essential that any non-shot noise contributions to image background are considered and quantified carefully, given their influence on measurement sensitivity and precision.

What is most encouraging, however, is that there appears substantial scope for improvement that will enable quantitative characterization of complex mixtures of biomolecules with a resolution and sensitivity that covers almost all species and interactions. Moreover, implementation of approaches that enable extended observation of individual molecules either in nanochannels^4^ or on bilayers^13,34^ could bring about even further improvements, much in the spirit of the advances brought about by similar strategies in mass spectrometry, such as charge detection and orbitrap mass spectrometry.^35,36^ Alternatively, if high resolution and sensitivity are not required, integration times can be drastically reduced, which will enable measurement at higher analyte concentrations, providing access to a broader range of affinities and ultimately weak interactions, further broadening the application scope of MP for characterizing biomolecular interactions and dynamics.

## 4 Methods

### 4.1 Description of Image Formation

We model image formation in MP as a 3D (complex-valued) convolution of electric fields (reference and scattered) with an APSF,^27^ which is implemented as a multiplication in Fourier space (through the convolution theorem). In contrast to previous reports on the computation of such an interferometric PSF,^15,16^ we employ a Fourier-based approach (see “slice propagation method” in ref (37)), with the benefits of being flexible and fast. This is mainly due to the fact that the necessary integrations over the BFP distribution (in refs (15) and (16)), is replaced by fast-Fourier transforms (FFTs) while also enabling simple modifications of the complex amplitude transmission in the pupil of the imaging system (i.e., no radial symmetry required). Propagating the electric fields along the optical axis is achieved through the angular spectrum method,^38^ where typical wrap-around effects are being avoided by replacing the FFT through a chirp-Z transform, as shown in ref (39). The strength of the reference and scattered fields are given by Fresnel’s coefficients *r* and *t* (reflection and transmission) at a glass–water interface and by the scattering coefficient *s* of a small spherical particle, in the Rayleigh regime.^14^ The scattering coefficient *s*, is related to the polarizability α, i.e., to the volume *V* of the spherical scatterer and the refractive indices of the protein *n*_p_ and the surrounding medium *n*_m_. High NA focusing effects are included by taking into account the refraction of light toward the nominal focus,^27^ the aplanatic factor,^37^ and the additional Gouy phase shift of the scattered light^15^ (all summarized in the *h⃗*-term). The influence of phase aberrations due to the refractive index mismatch at the coverslip interface is described using the Gibson–Lanni model.^40^ The scattering of the protein at the water–glass interface is given as a distribution at the back-focal plane of the objective,^41^ which further enables us to add a mask that attenuates and/or delays the reference component.^9^ A more detailed description of the underlying theoretical framework is given in Sections S1–S8.

### 4.2 Simulation Parameters

All numerical results assume a 1.42 NA oil-immersion (*n*_i_ = 1.515) objective while imaging at a wavelength of λ = 445 nm. The data shown in Figure 1 corresponds to an effective pixel size of 0.057 μm with 70 × 70 pixels, yielding a field-of-view (FoV) of 4 μm × 4 μm = 16 μm^2^. The step size along the axial direction was chosen to match the lateral size, ±2 μm propagation from the nominal focus. In case of the simulated landing assay data (all remaining figures), the effective pixel size was 70 nm at 128 × 128 pixels, resulting in a FoV of ∼80 μm^2^ while only computing a single in-focus slice. In this case, we set the attenuation mask to add a π/2 phase delay for , which results in phase-matching of the two fields at the detector and optimum contrast at the nominal focal plane (see Sections S5 and S12). The illumination was set to be (right-) circularly polarized (simulated as the average of azimuthally distributed linear polarizations), except for Figure 3b,c where we defined it to be linearly polarized. In terms of illumination intensity , we directly defined the detected photons at the detector, omitting the need to specify the overall efficiency of the detection system (including QE of the detector, losses at the optics, etc.). For the landing assay simulations, this yielded in 10^6^ photons per 70 nm pixel per 500 μs exposure time, which corresponds to the 10^7^ photoelectrons mentioned earlier, when performing the ratiometric calculation with *N* = 100. The simulations were run on a personal computer (Windows 10; Intel Core i7-6700 CPU @ 3.40 GHz; 16 GB RAM) and required ∼20 μs per simulated voxel (tested on a 256 × 256 × 256 grid).

### 4.3 General Measurement Routine

Data were either collected on a TwoMP (Refeyn, UK) or on a custom-built mass photometer. Coverslips (Menzel-Glaeser, 24 × 50 mm # 1.5 SPEZIAL; Thermo Fisher Scientific, U.S.) were cleaned to remove any contaminants by sonication for 5 min in 50/50 isopropanol and Milli-Q water (Merck, Germany), followed by 5 min in Milli-Q only. They were then dried using N_2_ and stored in a covered box to prevent re-contamination. Immediately before measurement, a silicon gasket (Grace Bio-Labs CultureWell, 3 × 1 mm; U.S.) was laid on the coverslip to contain the sample. The coverslip was placed on a sample-stage (*xyz* for TwoMP; *z*-only for custom-built system) above the objective and a small amount of immersion oil (Zeiss, Immersol 518 F; Germany) was added between the coverslip and objective to form a continuous interface. Once the gasket and objective were aligned, buffer was loaded into the gasket using a micropipette (Gilson Pipetman, U.S.). In case of the TwoMP, this allowed the focus position of the setup to be found and the autofocus to be set before the protein began binding to the coverslip. The custom-built system was operated without an autofocus but proved to be stable enough over the recording time. Once focus had been set, the protein was diluted in an Eppendorf tube (Eppendorf, 1.5 mL; Germany) to give 20 μL of the sample, at a concentration of 10–50 nM. The sample was added to the gasket and the focus quickly rechecked. If the added sample had been kept on ice, the refractive index of the solution could change when the preloaded buffer and sample were mixed due to the difference in temperature, which changed the focus position slightly. A movie was then recorded. The protein dynamin-1 ΔPRD was used as a mass calibrant. It is highly stable, easily produced in large volumes, and oligomeric, providing a large number of species of known mass (sometimes up to 7) with which to calibrate, increasing the accuracy of the calibration.

### 4.4 Materials

Reagents used were from Sigma-Aldrich (U.S.), unless otherwise stated. Water was ultrapure Milli-Q (Merck, Germany), and all solutions were filtered through a 0.2 μm filter (Millipore, U.S.) before use.

The disordered protein SUMO-SRSF1 containing the solubilizing mutations Y37S and Y72S^42^ was cloned by Gibson assembly into a pET28 plasmid (kind gift of B. B. Kragelund, University of Copenhagen, Denmark) downstream of a His_6_-SUMO tag. The construct was transformed into chemically competent C41 *Escherichia coli* (Lucigen). Cultures were grown in 2x YT medium supplemented with 50 μg/mL kanamycin at 37 °C until an OD_600_ of ∼1.5 was reached, and protein expression was induced using 0.5 mM IPTG at 20 °C overnight. Cells were harvested, resuspended in buffer A (20 mM sodium phosphate pH 7.5, 800 mM NaCl, 5% glycerol, 0.01% Tween-20, 2 mM dithiothreitol (DTT), 150 mM l-arginine, and 150 mM l-glutamate) supplemented with 10 mM MgCl_2_, 10 U/mL benzonase (Merck), and 20 μg/mL RNase A (NEB), and lysed by passing the suspension three times through a high-pressure homogenizer (HPL6, Maximator) cooled to 4 °C at 15,000–20,000 psi. The lysate was clarified by centrifugation and applied to a HisTrap Excel column (Cytiva, 5 mL per 1 L cell culture) equilibrated in buffer A. The column was washed with 15 column volumes (CVs) of buffer A, followed by 7 CVs buffer B (20 mM sodium phosphate pH 7.5, 3 M NaCl, 5% glycerol, 0.01% Tween-20, 2 mM DTT, 100 mM l-arginine, and 100 mM l-glutamate) and 7 CVs of 97% buffer A and 3% buffer C (20 mM sodium phosphate pH 7.5, 800 mM NaCl, 5% glycerol, 0.01% Tween-20, 2 mM DTT, 150 mM l-arginine, 150 mM l-glutamate, and 500 mM imidazole), before elution with buffer C. Fractions containing protein were pooled and diluted at least 8-fold with buffer D (20 mM HEPES pH 8.0, 10% glycerol, 0.001% Tween-20, 0.5 mM Tris-(2-carboxyethyl) phosphine, and TCEP) and 5 M NaCl until the sample was clear (∼0.8 M ionic strength or 55 mS/cm). Nucleotides and protein contaminants were removed from the sample by ion exchange chromatography using a MonoS 5/50 GL column (Cytiva) and a gradient of 30–70% of buffer E (20 mM HEPES pH 8.0, 2 M NaCl, 10% glycerol, 0.01% Tween-20, and 0.5 mM TCEP). Fractions with absorbance ratios of A260/A280 < 0.7 were pooled, flash frozen in liquid nitrogen, and stored at −80 °C. Starmaker was prepared as described previously.^31^

Stock solutions of SUMO-SRSF1 were at 29 μM protein. 1 μM aliquots were prepared in 20 mM HEPES (pH 7.4), 1 M NaCl, 1 mM DTT, and 5% glycerol buffer and flash frozen. High salt was required to stabilize the protein. DTT is a reducing agent, preventing oligomerization via disulfide bonds as SUMO-SRSF1 contains two internal cysteines. Starmaker was diluted in 20 mM Tris (pH 8.4) and 50 mM KCl. The concentration was unknown, so measurements of a range of dilutions from the original stock were taken to estimate the concentration. For Starmaker, coverslips were positively charged with APTES. Coverslips were cleaned as described before, then plasma cleaned for 8 min. The coverslips were washed in acetone and submerged in a 2% APTES/acetone solution. After 2 min, the coverslips were washed with acetone again and placed in an oven for 1.5 h at 110 °C. Finally, the coverslips were sonicated for 5 min in isopropanol, then water, and dried under N_2_.

### 4.5 Data Analysis

For the analysis of the recorded data, we used an in-house python package. Raw frames from the measurement *I*_*i*_ were imported and converted into ratiometric frames, by averaging two stacks of *n*_avg_ raw frames;  from frame (*i*) to (*i* + *n*_avg_) and  from frame (*i* + *n*_avg_ + 1) to (*i* + 2*n*_avg_ + 1)
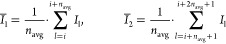
21

Those two stacks are then used to compute the relative difference
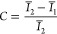
22

This eliminates the background signal (from the glass roughness) that is constant throughout the measurement. Protein binding events that occur during the measurement are not constant and hence are not removed when computing the relative difference. They appear as spots that fade in and out of the image as the protein binds and then becomes part of the background. Once the ratiometric frames have been calculated, protein binding events are identified by filtering the detected events for groups of pixels that meet a minimum radial symmetry and for pixels with a minimum signal above the background noise. A PSF (either a theoretical^3^ or experimental model) is then fitted to each event to determine its contrast. The use of an experimental PSF is necessary when analyzing data that corresponds to illuminating the scatterer with linearly polarized light (e.g., in case of the DNA measurements). To obtain the experimental PSF, the initial PSF detection parameters (found with the theoretical model) are used to align the cropped images (typically 7 × 7 pixels) of the found landing events, by employing a cubic spline interpolation. Those sub-images are then averaged together, while outliers (Pearson’s correlation test) are being removed from this average. The resulting cubic spline model is then used to determine the contrast of each landing event. The obtained contrast values for all events are then plotted as a mass histogram. For a particular species, the event contrasts are usually normally distributed allowing a Gaussian to be fitted to the respective peaks. The Gaussian position, width, and area were used to characterize the contrast of each peak μ, standard deviation σ, and counts, respectively. To generate a mass calibration, this procedure was applied to a measurement of dynamin-1 ΔPRD. The peak contrasts were used for calibration by plotting against the corresponding species mass. In case of the simulated data, the calibration was performed against the first four oligomeric states of BSA shown in Figure 2b while deliberately reducing the numerically applied shot noise to obtain an accurate calibration.

### 4.6 Measurements of Double-Stranded DNA

Data were taken on both a custom-built MP setup that uses 465 nm linearly polarized illumination and a TwoMP (Refeyn, UK) with 488 nm circularly polarized illumination. APTES coverslips with silicone gaskets were prepared via the procedure described above. 200, 400, and 600 base pair double-stranded DNA were prepared using standard procedures.^43^ For the MP measurements 200, 400, and 600 bp dsDNA were diluted to 3, 4, and 4 nM, respectively, in phosphate buffered saline (PBS). 20 μL of the sample was added to a gasket containing 5 μL of buffer. Data were acquired for 120 s following sample refocusing. The contrast values were converted into mass using a calibration curve obtained from a measurement of dynamin-1 ΔPRD (adjusted by a factor ×1.25, as described in Section S14). Note that the 600 bp dsDNA was excluded from being presented in this work (see Figure 3a) due to its length being beyond the diffraction limit of the detection system, which yields additional mass broadening. For completeness, however, it is presented in Section S14.

For the custom-built linearly polarized MP setup, data were acquired with the following parameters: 959 μs exposure time, 787 fps, 3.4 × 11.7 μm^2^ field of view, 3 × 3 pixel binning, and 4-fold temporal averaging. For the TwoMP, data were acquired with the following parameters: 1380 μs exposure time, 698 fps, 2.7 × 10.9 μm^2^ field of view, 6 × 6 pixel binning, and 4-fold temporal averaging. Both datasets were analyzed such that the ratiometric window size amounted to ∼50 ms.

### 4.7 Measurements of Partially and Fully Unfolded Proteins

Data were taken using a TwoMP mass photometer (Refeyn, UK) and analyzed using a custom-written Python package, based on the procedure described in Young et al.^3^ Coverslips (Menzel-Gläser, 24 × 50 mm # 1.5 SPEZIAL; Thermo Fisher Scientific, U.S.) were cleaned, a silicon gasket (Grace Bio-Labs CultureWell, 3 × 1 mm; U.S.) was laid on top, and 4 μL of buffer medium was added. Next, the protein was diluted in an Eppendorf tube (Eppendorf, 1.5 mL; Germany) to give 20 μL of sample and added into the gasket. Movies containing ∼1000–5000 binding events were recorded (60 s), analyzed, and converted into mass using a calibration curve (generated from a measurement of the oligomeric peaks of dynamin-1 ΔPRD).

SUMO-SRSF1 was diluted to 20 nM in a buffer of 20 mM HEPES (pH 7.4), 1 M NaCl, 1 mM DT, and 20 mM NaCl. A 20 mM Tris (pH 7.4) and 50 mM NaCl buffer was used to dilute Starmaker, allowing for a reduction or an increase in salt concentration upon measurement. For both proteins, 4 repeats were taken at each condition, with no significant unbinding in any repeat.

### 4.8 PDB IDs of Several Analyzed Proteins

A list containing PDB IDs of the respective proteins shown in Figure 4a,b is displayed in Table 1.

**Table 1:** PDB IDs for Different Proteins Used to Investigate the Linear Relationship between the Protein Mass and Scattering Strength (See Figure 4a,b)

Name	HasA	Maspin	Strep.	BSA	Cyt-BC1	Hsp16.5	Myosin-V	Chloro	GroEL	IBDV
PDB ID	1B2V	1XQJ	4BX6	3V03	1BE3	1SHS	2DFS	6FKI	1GR5	2GSY

### 4.9 Enhancement Factor Describing the Scattering near a Glass–Water Interface

Table 2 reports the enhancement factor γ that describes the additionally detected scattering which corresponds to the near-field of a scatterer at the glass–water interface. Details on the computation of γ are given in Section S10.

**Table 2:** Enhancement Factor Describing the Scattering of a Dipole at the Glass–Water Interface for Different NAs

NA	1.2	1.3	1.4	1.5
*n*_*i*_	1.515	1.515	1.515	1.515
γ	1.14	1.21	1.53	1.67

## Supporting Information Available

Derivation of the underlying theoretical description and simulation routine (PDF)

## Notes

Data that supports the findings of this study are available from the authors upon request.

## Notes

An initial version of this manuscript has been made available as the following pre-print: Jan Becker, Jack S. Peters, Ivor Crooks, Seham Helmi, Marie Synakewicz, Benjamin Schuler, Philipp Kukura; A quantitative description for optical mass measurement of single biomolecules; 2023; 2023.03.28.534430; bioRxiv; doi: https://doi.org/10.1101/2023.03.28.534430; https://www.biorxiv.org/content/10.1101/2023.03.28.534430v1.full (accessed 31st May 2023).
